# Multi-Layered Antidepressant Mechanisms of Gami-Soyosan in a Corticosterone-Induced Mouse Model

**DOI:** 10.4014/jmb.2509.09033

**Published:** 2026-01-26

**Authors:** Seung-Ho Seo, Seungeun Yeo, Sang-Mi Kang, Yang-Hee You, Siwon Kang, Yujin Lee, Youngshik Choe, Chang-Su Na

**Affiliations:** 1 College of Korean Medicine, Dongshin University, Naju 58071, Republic of Korea; 2 Developmental Disorders & Rare Diseases Research Group, Korea Brain Research Institute, Daegu 41062, Republic of Korea

**Keywords:** Herbal medicine, Gami-Soyosan, Corticosterone-induced depression, Metabolomics, Oxidative stress

## Abstract

This study aimed to evaluate the antidepressant-like effects of Gami-Soyosan (GSS) in a corticosterone-induced mouse model of depression. Behavioral assessments, including the forced swim test and tail suspension test, demonstrated that GSS significantly reduced immobility, indicating improved coping behaviors comparable to those of fluoxetine. Serum analysis revealed that GSS lowered pro-inflammatory cytokines such as IL-6, IL-1β, and TNF-α, indicating an attenuation of systemic inflammatory cytokine responses in this model. Metabolomic profiling further showed that GSS modulated amino acid and nitrogen-related pathways, including branched-chain amino acids, arginine, and histidine/β-alanine metabolism, supporting the restoration of metabolic homeostasis under stress. Distinct metabolic signatures were also observed when compared to fluoxetine, indicating that GSS may exert antidepressant-like effects through partially different mechanisms. In addition, *in vitro* experiments using neuronal cells demonstrated that GSS attenuated oxidative stress by reducing whole-cell ROS generation and enhancing lysosomal activity, highlighting a neuroprotective role. Together, these findings provide multi-layered evidence that GSS acts through behavioral, immune, metabolic, and oxidative pathways, supporting its potential as a complementary therapeutic approach for stress-related depression.

## Introduction

Depression is a widespread psychiatric disorder that significantly impairs emotional and cognitive function while imposing a heavy socioeconomic burden [1]. Although pharmacological treatments such as selective serotonin reuptake inhibitors (SSRIs) and serotonin-norepinephrine reuptake inhibitors (SNRIs) are commonly prescribed, their efficacy is often limited by delayed therapeutic onset, side effects, and inter-individual variability in patient response [2-5]. As a result, there has been growing interest in traditional herbal medicine as a complementary approach. In particular, traditional East Asian multi-herbal formulations have gained attention for their potential to address the multifaceted pathophysiology of depression [6, 7]. These formulations, composed of various medicinal herbs, are hypothesized to act on multiple biological targets such as neurotransmitter systems [8, 9], hormonal regulation [10, 11], and immune modulation [12], although further scientific validation is warranted.

Herbal prescriptions are often valued in traditional medicine for their synergistic therapeutic effects. Their chemically diverse constituents allow simultaneous targeting of multiple pathological pathways, offering both short-term and long-term benefits in the prevention and treatment of complex diseases [13]. One such prescription is Gami-Soyosan (GSS), a traditional formula widely used in East Asian medicine to manage neuropsychiatric symptoms, including anxiety, irritability, and insomnia [14]. In Korea, GSS is covered by the National Health Insurance Service for treatment of conditions such as neurosis and phobic anxiety disorder [15]. A growing body of clinical and experimental research supports the potential antidepressant, anti-stress, and antioxidant properties of GSS [16-18]. However, despite these promising findings, the underlying biological mechanisms of GSS remain poorly understood. In particular, few studies have explored its systemic effects using omics-based approaches, such as metabolomics, which could provide comprehensive insight into its therapeutic actions.

Metabolomics, a core discipline within systems biology, enables the comprehensive profiling of small molecule metabolites that reflect dynamic physiological states [19]. This approach has proven particularly valuable in the field of neuropsychiatric research, where complex, multi-layered biological processes contribute to both disease pathogenesis and therapeutic outcomes [20]. Metabolomics may serve as a valuable tool for exploring the systemic effects of multi-herbal prescriptions like GSS, offering the potential to identify metabolic alterations and gain insights into mechanisms related to their antidepressant properties.

To address this knowledge gap, our study investigated the antidepressant effects of GSS in a mouse model of depression and to explore its potential mechanisms of action, focusing on untargeted metabolomic analysis to identify key metabolic pathways.

## Materials and Methods

### Preparation of GSS and Reagents

GSS was purchased from Hanpoong Pharm & Foods Co., Ltd. (Republic of Korea). Corticosterone (CORT), used to induce a depression-like state in mice, was purchased from Sigma-Aldrich Korea (Republic of Korea). Fluoxetine (Sigma-Aldrich Korea), a widely used antidepressant, was used as a positive control in the experimental procedures.

### Animal Experiment

**Fig. 1:**
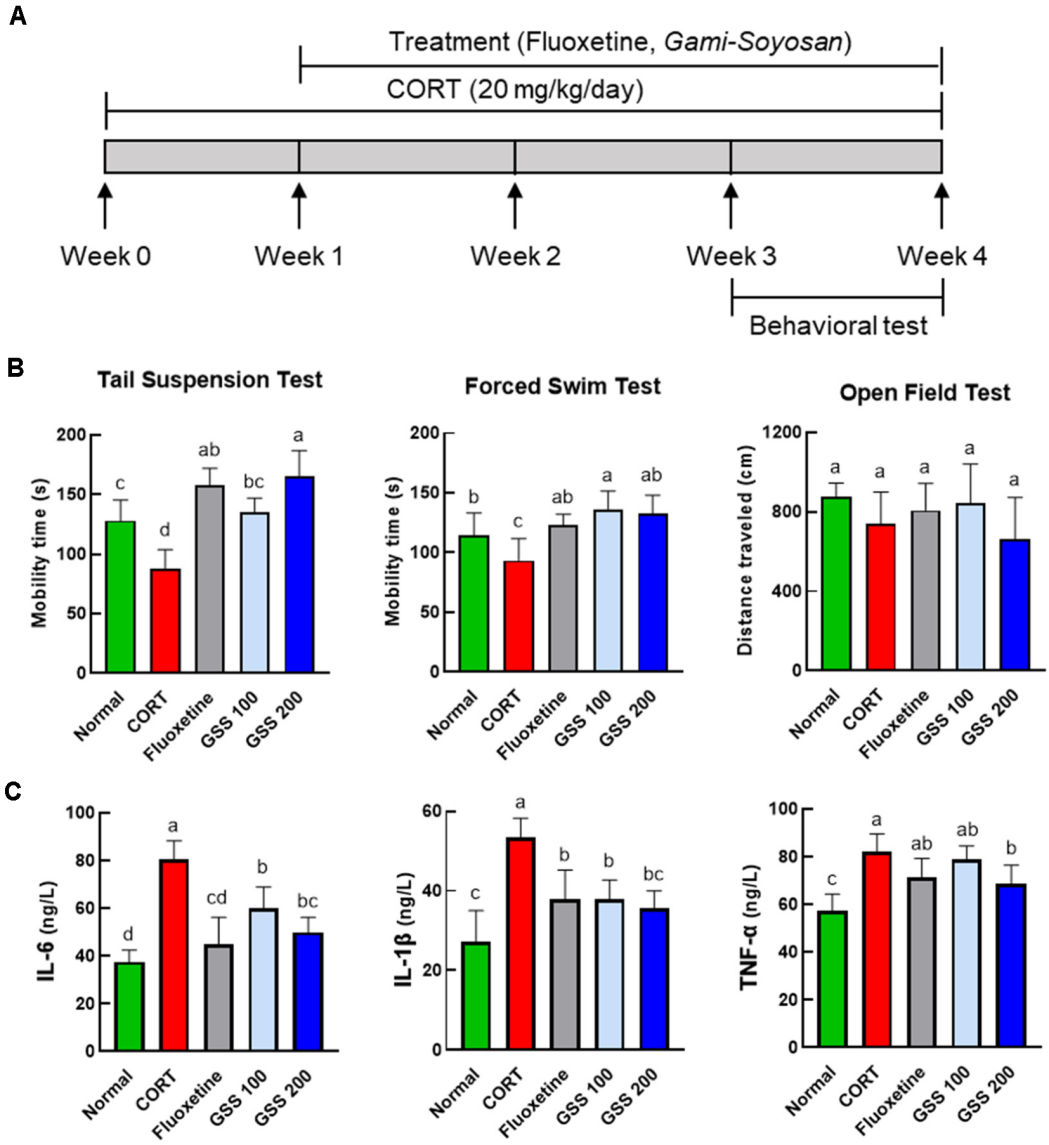
Experimental design, behavioral outcomes, and cytokine levels in corticosterone-induced depression model. Five experimental groups were included: normal control (Normal), corticosterone-treated (CORT), fluoxetine-treated after corticosterone administration (Fluoxetine), Gami-Soyosan (GSS) at 100 mg/kg after corticosterone administration (GSS 100), and GSS at 200 mg/kg after corticosterone administration (GSS 200). (**A**) Schematic representation of experimental design. (**B**) Behavioral results of the TST, FST, and OFT. (**C**) Serum cytokine levels measured by ELISA. Data are expressed as mean ± standard error of the mean (SEM). Statistical analysis was performed using one-way analysis of variance (ANOVA) followed by Tukey’s post hoc test.

All animal procedures were approved by the Institutional Animal Care and Use Committee of Dongshin University (DSU-IACUC, Approval No. DSU2024-06-01). Seven-week-old male C57BL/6 mice were purchased and housed under specific pathogen-free (SPF) conditions with a 12-hour light/dark cycle, controlled temperature (20 ± 2°C), and relative humidity (60 ± 5%). After a 7-day acclimatization period, the mice were randomly assigned into five groups (*n* = 8 per group): Normal group (0.9% saline, administered orally), CORT group (20 mg/kg/day, administered by subcutaneous injection), Fluoxetine group (15 mg/kg/day, administered orally), GSS 100 group (100 mg/kg/day, administered orally), GSS 200 group (200 mg/kg/day, administered orally). The group size of eight mice per group was determined with reference to previous studies using mouse depression models with similar behavioral and biochemical endpoints and in accordance with the principle of using the minimum number of animals necessary to obtain statistically meaningful results under our institutional animal ethics guidelines [21]. The experiment was conducted over 21 days, excluding the acclimatization period. CORT was administered by subcutaneous injection daily between 7:00–8:00 AM, while fluoxetine and GSS were administered orally once daily. Behavioral tests were performed during the final phase of the experiment: the open field test on day 18, the forced swimming test on day 19, and the tail suspension test on day 20. All behavioral assessments were conducted at 3:00 PM to minimize diurnal variation. The overall experimental timeline and treatment protocol are illustrated in Fig. 1A. On the final day of the experiment, mice were euthanized by inhalation of isoflurane, and their body weights were recorded. Blood samples were collected by cardiac puncture, and serum was separated by centrifugation at 12,000 rpm for 15 min. The obtained serum was divided for metabolomic profiling and cytokine analysis. All serum samples were stored at −80°C until further use.

### Open Field Test (OFT)

The OFT was conducted with reference to the protocol described by Song *et al*. [22]. Each mouse was placed individually in the center of an open-field box (30 × 30 × 40 cm), with the floor divided into nine equal sections. Locomotor activity was recorded for 5 min and analyzed using ANY-maze software (Stoelting, USA). The total number of sections crossed was used as an indicator of horizontal movement. To eliminate residual odors that could affect behavior, the apparatus was thoroughly cleaned with 75% ethanol between trials.

### Forced Swimming Test (FST)

The FST was performed with reference to a previously reported method, with minor modifications [23]. Each mouse was placed individually into a transparent glass cylinder (height: 30 cm; diameter: 18 cm) filled with water to a depth of 20 cm, maintained at 24 ± 2°C. The test lasted for 6 min, during which the first 2 min were considered an adaptation period, and immobility time was recorded during the final 4 min. Mice were considered immobile when they ceased active movement and only made minimal motions to keep their heads above the water. The test was conducted in a quiet room with consistent lighting conditions. After each trial, the animals were removed, dried, and returned to their home cages, and the water was replaced.

### Tail Suspension Test (TST)

The TST was performed following the protocol of Li *et al*. [24]. Mice were suspended by the tail using adhesive tape placed approximately 1 cm from the tip, and positioned at a height of 5 cm above the floor of a testing chamber (25 × 25 × 30 cm). Each session lasted for 6 min, with the first min considered an adaptation period and immobility time recorded during the remaining 5 min. Mice were defined as immobile when they hung passively and remained completely motionless. All tests were carried out under quiet conditions to minimize environmental stress.

### Enzyme-Linked Immunosorbent Assay (ELISA)

Serum samples were obtained by centrifugation at 3500 rpm for 10 min. Hippocampal tissues were homogenized in 0.9% physiological saline and centrifuged at 12,000 rpm for 10 min at 4°C to collect the supernatant. The concentrations of interleukin-6 (IL-6), interleukin-1β (IL-1β), and tumor necrosis factor-α (TNF-α) in serum and hippocampal extracts were measured using commercial ELISA kits following the manufacturer’s instructions. Absorbance was recorded at 450 nm using a microplate reader.

### Ultra-Performance Liquid Chromatography–Quadrupole Time-of-Flight Mass Spectrometry (UPLC-QTOF-MS) Analysis

Serum metabolomic profiling was conducted using a Waters ACQUITY UPLC I-Class system (Waters Corp., USA) coupled with an Xevo G3 QTOF mass spectrometer. Chromatographic separation was performed on both an ACQUITY UPLC BEH C18 column (2.1 × 100 mm, 1.7 μm particle size) and an ACQUITY UPLC HSS T3 column (2.1 × 100 mm, 1.8 μm particle size). For the C18 column, the column temperature was maintained at 40°C, and the mobile phase consisted of water (solvent A) and acetonitrile (solvent B), both containing 0.1% formic acid. A linear gradient was applied at a flow rate of 0.4 ml/min as follows: 98% A (0–0.25 min), 98–2% A (0.25–10 min), 2% A (10–13 min), 2–98% A (13–13.01 min), and 98% A (13.01–15 min). For the HSS T3 column, the flow rate was also set to 0.4 ml/min, with the following gradient program: 95% A (0–3 min), 95–50% A (3–8 min), 50–5% A (8–12 min), 5% A (12–14 min), and 95% A (14–20 min). The injection volume was 2 μl per sample.

The desolvation gas flow rate and temperature were set at 1,000 L/h and 500°C, respectively. Cone gas flow was maintained at 150 L/h, and the ion source temperature was set to 120°C. The capillary voltage was set to 2.8 kV in positive mode and 2.5 kV in negative mode, with a cone voltage of 40 V.

In the data-independent MS^E^ acquisition mode, the mass range was set from m/z 50 to 1,200 Da with a scan duration of 0.1 sec. Sodium formate served as the calibration solution to ensure mass accuracy. For real-time mass correction, leucine enkephalin at a concentration of 200 pg/ml (m/z 556.2771 in ESI+ and m/z 554.2620 in ESI−) was continuously introduced as the lock mass, which was set for real-time calibration. The mass resolution was adjusted to 10,000 full width at half maximum (FWHM). Argon was applied as the collision gas in the transfer Triwave collision cell, with the collision energy ramped from 20 to 40 eV. To verify system stability and achieve column equilibration, ten quality control (QC) samples were analyzed prior to the sequence. During the analysis, a blank sample and a QC sample were injected after every ten study samples to monitor stability throughout the run. The QC samples were prepared by mixing 20 μl aliquots from each extracted sample.

### Data Processing

For untargeted metabolomics analysis, raw data files generated by MassLynx software (v4.1, Waters Corp.) were imported into Progenesis QI (Non-linear Dynamics, UK). Feature alignment was performed using the full MS scan of QC samples as a reference, followed by peak detection, deconvolution, and normalization across all samples. For other metabolites, proposed structures were inferred from their fragmentation patterns. To account for differences in sample concentration and injection volume, all spectral intensities were normalized to the total ion current (TIC) of each sample. Compound identification was based on accurate mass measurements within a 10 ppm tolerance and MS/MS fragmentation data, using the Human Metabolome Database (HMDB; http://www.hmdb.ca/) and the MassBank of North America (MoNA; https://mona.fiehnlab.ucdavis.edu/) as the primary annotation sources. To visualize the variance in the UPLC-QTOF-MS data, orthogonal projection to latent structure discriminant analysis (OPLS-DA) was performed using SIMCA-P 17.0 (Umetrics, Sweden). Cross-validation was conducted using a permutation test repeated 200 times. Metabolites with a variable importance in projection (VIP) score greater than 1.0 in the OPLS-DA model and a false discovery rate (FDR)-adjusted *p*-value < 0.05 in one-way ANOVA were identified as characteristic discriminatory metabolites. The potential characteristic metabolites were identified based on VIP values obtained from orthogonal comparisons. Metabolites with VIP values greater than 1.0 and *p*-values less than 0.05 were identified as characteristic discriminatory metabolites. Hierarchical clustering heatmaps of serum metabolites were generated using autoscaled data. Euclidean distance was applied as the distance metric, and Ward’s method was used for clustering. Group visualization was performed based on group averages. The metabolites identified as potential biomarkers were subjected to pathway analysis using MetaboAnalyst 6.0 [25]. Pathways with *p*-values less than 0.05 were considered significantly enriched and defined as the major pathways. Data scaling was performed using auto scaling. The distance measure was set to Euclidean, the clustering method was Ward, and the group visualization was set to show only the group average.

### Correlation Analysis between Metabolites, Cytokines, and Behavioral Outcomes

Correlation analyses between serum metabolites, cytokines, and behavioral outcomes were performed in MetaboAnalyst 6.0. Immobility times in the FST and TST, locomotor activity in the OFT, serum cytokine levels (IL-6, IL-1β, and TNF-α), and normalized serum metabolite intensities were combined into a single data matrix. All variables were autoscaled, and pairwise Spearman’s rank correlations were calculated. Correlation structure was visualized as a clustered correlation heatmap. Correlation pairs with an FDR-adjusted *p*-value < 0.05 and an absolute Spearman correlation coefficient ≥ 0.7 were additionally summarized in a separate table.

### HT22 Cell Culture and CCK-8 Viability Assay

The mouse hippocampal HT22 cell line was originally obtained from the American Type Culture Collection (ATCC, USA). Cells were maintained in Dulbecco’s Modified Eagle Medium (DMEM) supplemented with 10% (v/v) fetal bovine serum (FBS), 100 U/ml penicillin, and 100 μg/ml streptomycin in a humidified incubator (5% CO_2_, 37°C). Cells were passaged when they reached approximately 70% confluency. For experiments, 4.8 × 10^5^ cells were seeded into each well of a 96-well plate and cultured overnight.

Cells were pretreated with modified GSS at concentrations of 50, 100, or 200 μg/ml for 2 h, followed by exposure to 300 μM hydrogen peroxide (H_2_O_2_; Sigma-Aldrich, USA) in the presence or absence of GSS for an additional 24 h. For the cell viability assay, the culture medium was replaced with fresh medium containing H_2_O_2_, GSS, and 0.1 volume of CCK-8 solution (Dojindo Laboratories, Japan). After a 3-h incubation, absorbance was measured at 450 nm using a FlexStation 3 multi-mode microplate reader (Molecular Devices, USA).

### Assays for Mitochondrial and Lysosomal Activities, and Cellular or Mitochondrial Reactive Oxygen Species

HT22 cells were pretreated with 100 μg/ml GSS for 2 h, followed by incubation with 300 μM H_2_O_2_ and 100 μg/ml GSS for an additional 24 h. After washing with phosphate-buffered saline (PBS), the cells were incubated at 37°C for 30 min with 500 nM MitoSOX (Thermo Fisher Scientific, USA) to measure mitochondrial reactive oxygen species (ROS) and 250 nM MitoTracker (Thermo Fisher Scientific) to assess mitochondrial activity.

MitoSOX and MitoTracker fluorescence signals were measured at excitation/emission wavelengths of 396/610 nm and 490/516 nm, respectively, using a FlexStation 3 multi-mode microplate reader (Molecular Devices). For the assessment of cellular ROS and lysosomal activity, cells were incubated at 37°C for 30 min with 5 μM CellROX (Thermo Fisher Scientific) and 50 nM LysoTracker (Thermo Fisher Scientific). CellROX fluorescence was measured at 640/665 nm using the FlexStation 3, and LysoTracker signals were imaged at 504/551 nm using a Nikon A1Rsi/Ni-E confocal microscope (Nikon, Japan) and analyzed with NIS-Elements AR software (Nikon). To normalize fluorescence signals from MitoSOX, MitoTracker, and CellROX staining, cells were co-stained with 10 μM Hoechst 33342 (Thermo Fisher Scientific), and fluorescence was measured at 350/461 nm using either the FlexStation 3 or the Nikon confocal microscope.

### Measurement of Total Glutathione and Catalase Activity

HT22 cells were pretreated with GSS (100 μg/ml) for 2 h, followed by exposure to H_2_O_2_ (300 μM). Twenty-four hours after H_2_O_2_ treatment, cells were harvested, washed, and resuspended in ice-cold 1× PBS. The cell suspensions were sonicated on ice, and the lysates were clarified by centrifugation at 12,000 rpm for 10 min at 4°C. The resulting supernatants were analyzed according to the manufacturers’ protocols using the OxiSelect Total Glutathione (GSSG/GSH) Assay Kit (Cell Biolabs; Cat. #STA-312) and the OxiSelect Catalase Activity Assay Kit (Cell Biolabs; Cat. #STA-341).

### Immunofluorescence Cell Imaging

HT22 cells were fixed with 4% paraformaldehyde for 30 min at room temperature, then permeabilized and blocked in PBS containing 0.5% Triton X-100 and 10% bovine serum albumin (BSA) for 1 h at room temperature. Cells were incubated with primary antibodies diluted 1:100 in blocking buffer (anti-TFEB; Novus Biologicals, NBP2-41167; anti-Nrf2; Thermo Fisher Scientific, PA5-27882) for 3 h at room temperature. After washing with PBS containing 0.2% Tween-20 (PBST), cells were incubated with Alexa Fluor 555–conjugated goat anti-rabbit IgG (H+L) cross-adsorbed secondary antibody (1:250 dilution) for 1 h at room temperature in the dark. Nuclei were counterstained with Hoechst 33342 (Thermo Fisher Scientific, #62249).

Immunofluorescence images were acquired using a Nikon A1 Rsi/Ni-E confocal microscope equipped with laser lines at 403 nm, 457/488/514 nm, 561 nm, and 640 nm. Alexa Fluor signals were detected to visualize the Nrf2 and TFEB. Hoechst signals were collected using a 450/50 nm excitation and 400/50 nm emission filter set. Images were analyzed with NIS-Elements AR software (Nikon).

### Western Blot Analysis

Protein concentrations were determined using a BCA protein assay kit (Thermo Fisher Scientific). Equal amounts of protein (15 μg per lane) were separated on 4–20% Mini-PROTEAN TGX precast gels (Bio-Rad Laboratories) and transferred to Immobilon-P PVDF membranes (Millipore). Membranes were blocked for 30 min at room temperature in PBS containing 5% (w/v) skim milk (BD Biosciences) and 0.1% Tween-20 (Merck Millipore). The membranes were then incubated overnight at 4°C with primary antibodies such as anti-Nrf2 (Thermo Fisher Scientific, PA5-27882), anti-Keap1 (Thermo Fisher Scientific, PA5-99434), and anti-GAPDH (OriGene, TA802519). After washing with PBST, membranes were incubated with HRP-conjugated secondary antibodies for 30 min at room temperature. Protein bands were detected using an enhanced chemiluminescence (ECL) reagent kit (Thermo Fisher Scientific) and imaged with a chemiluminescence detection system.

### Statistical Analysis

Statistical analyses were performed using GraphPad Prism 9 (GraphPad Software, USA) and SIMCA-P 17.0 (Umetrics). The distribution of each dataset was assessed using the D’Agostino–Pearson omnibus normality test. For behavioral, cytokine, and cellular assays involving more than two groups, data were analyzed using ordinary one-way ANOVA with a single pooled variance, followed by Tukey’s multiple comparisons test. For metabolomics data, univariate analysis of individual serum metabolites across the five experimental groups was performed using one-way ANOVA, and *p*-values were adjusted for multiple comparisons by controlling the FDR using the Benjamini–Hochberg procedure. Metabolites with an FDR-adjusted *p*-value < 0.05 were considered statistically significant, and a *p*-value < 0.05 was regarded as statistically significant for all other analyses.

## Results

### Effects of GSS on Depression-Like Behaviors in CORT-Induced Mice

While it is inherently challenging to replicate the complexity of human depression in animal models, a variety of preclinical paradigms have been developed to induce depression-like phenotypes [26]. One widely used approach involves the chronic administration of CORT in mice, which mimics the sustained elevation of glucocorticoid levels typically observed in stress-related disorders [27, 28]. Therefore, we employed this model to induce a consistent depression-like state in mice.

To assess the antidepressant-like effects of GSS, behavioral assessments were performed in a CORT-induced mouse model of depression. The behavioral assessments included the TST, FST, and OFT. As expected, in both the TST and FST (Fig. 1B), CORT-treated mice exhibited significantly reduced active coping behavior, a key indicator of depression-like symptoms. These results validate the CORT-induced depression-like model and provide a foundation for evaluating the therapeutic effects of GSS [29-31]. Treatment with fluoxetine and GSS (100 and 200 mg/kg) significantly reduced immobility in both the FST and TST compared to the CORT group. A dose-dependent effect was particularly evident in the TST. Unlike the TST and FST, which are specifically designed to assess behavioral despair and are highly sensitive to antidepressant effects, the OFT primarily reflects locomotor activity and anxiety-related behavior [32, 33]. In the OFT, exploratory activity was decreased following CORT administration and an increasing trend was observed following treatment with fluoxetine or GSS at 100 mg/kg even though the differences between groups were not statistically significant. The absence of statistically significant differences in the OFT may reflect the relatively lower sensitivity of this test to detecting depression-like behaviors, particularly in CORT-induced models, where behavioral changes are often more pronounced in measures of despair rather than in exploratory or locomotor activity.

### Effects of GSS on Serum Pro-Inflammatory Cytokines in CORT-Induced Mice

In addition to the behavioral changes, chronic CORT exposure significantly increased serum levels of IL-6, IL-1β, and TNF-α compared with the Normal group, confirming the presence of systemic inflammation (Fig. 1C). Treatment with fluoxetine or GSS attenuated these cytokine elevations. Both fluoxetine and GSS at 100 and 200 mg/kg significantly reduced IL-6 and IL-1β levels relative to the CORT group, whereas only GSS at 200 mg/kg significantly decreased TNF-α, with fluoxetine and GSS 100 mg/kg showing a non-significant trend toward reduction.

### Metabolomic Profiling of CORT-Induced Mice Following GSS Treatment

**Fig. 2:**
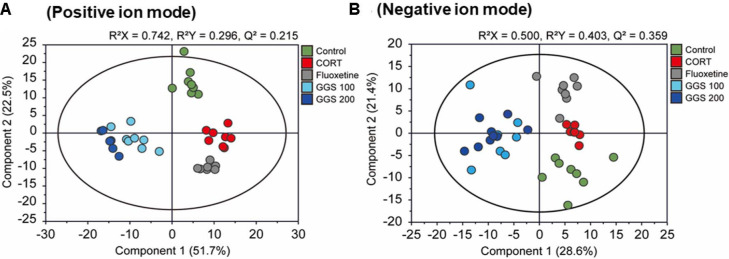
OPLS-DA score plots of serum metabolomic profiles from the five experimental groups based on UPLC-QTOF-MS in CORTinduced depression-like mice. The metabolic profiles of the five experimental groups were plotted using OPLS-DA scores in the positive (**A**) and negative (**B**) ion modes. In the positive ion mode, clearer separation among groups was observed, indicating distinct metabolic shifts induced by CORT and partial recovery after GSS or fluoxetine treatment. In the negative ion mode, group separation was less pronounced, suggesting weaker metabolic alterations in this mode.

Metabolic alterations following GSS administration in the CORT-induced mouse model were evaluated using OPLS-DA (Fig. 2). Clear separation among the five groups was observed in both ion modes, with particularly distinct clustering in the positive mode (R^2^X = 0.742, R^2^Y = 0.296, Q^2^ = 0.215) and acceptable model performance also in the negative mode (R^2^X = 0.500, R^2^Y = 0.403, Q^2^ = 0.359). A 200-iteration permutation test confirmed that the models were not overfitted (Fig. S1). Relative to the Normal group, CORT treated mice exhibited a markedly altered serum metabolic profile. Both GSS-treated groups and the fluoxetine group were clearly separated from the CORT group and also from each other, suggesting that GSS and fluoxetine modulate metabolic pathways through partly distinct mechanisms. Consistent with the behavioral data, no clear dose-dependent separation was observed between the GSS 100 and GSS 200 groups.

**Fig. 3:**
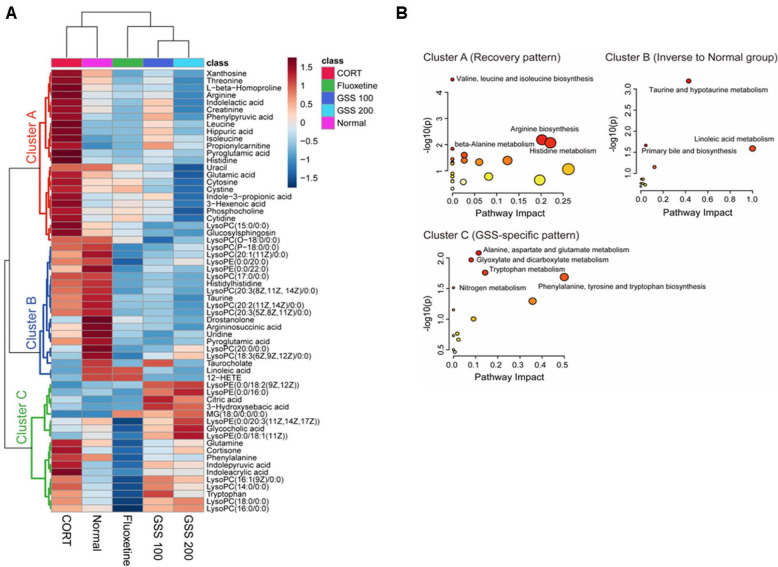
Hierarchical clustering and pathway analysis of serum metabolites altered by corticosterone induction and Gami-Soyosan (GSS) treatment. (**A**) Hierarchical clustering heatmap of serum metabolites that contributed to group separation in the OPLS-DA model, revealing three major clusters (Cluster A, Cluster B, and Cluster C) with distinct response patterns across the groups. Metabolites included in the heatmap were selected based on a VIP value greater than 1.0 in the OPLS-DA model and an FDR-adjusted *p*-value < 0.05 in one-way ANOVA across the five experimental groups. (**B**) Pathway analysis of metabolites in Clusters A–C. Cluster A shows a recovery pattern, with CORT-induced changes tending to return toward normal levels following GSS treatment; Cluster B displays an inverse pattern relative to the normal group, indicating treatment-induced reprogramming of selected metabolic pathways; Cluster C represents a GSS-specific pattern distinct from both fluoxetine and the normal recovery trend. All pathways shown are significantly enriched (*p* < 0.05).

To identify differentially expressed metabolites, those with VIP scores greater than 1.0 and statistical significance (*p* < 0.05) based on the OPLS-DA model were selected, and a clustered heat map was generated to visualize metabolite patterns across the five groups (Normal, CORT, Fluoxetine, GSS 100, and GSS 200) (Fig. 3A). Hierarchical clustering of the selected metabolites (VIP > 1.0, *p* < 0.05) revealed three distinct clusters, each characterized by unique response patterns across the experimental groups (Fig. 3A).

Pathway analysis was performed for each of the three metabolite clusters identified from the heatmap results (Fig. 3B). Cluster A comprised metabolites that exhibited marked alterations in the CORT group relative to the normal group, followed by a recovery trend in the treatment groups. These metabolites were primarily associated with valine, leucine and isoleucine biosynthesis, arginine biosynthesis, histidine metabolism, and beta-alanine metabolism. Cluster B contained metabolites that showed pronounced differences in the treatment groups compared with the normal group. This cluster was enriched in taurine and hypotaurine metabolism, primary bile acid biosynthesis, and linoleic acid metabolism. Cluster C consisted of metabolites that displayed clear differences between the fluoxetine group and the GSS-treated groups. This cluster was mainly linked to pathways such as alanine, aspartate and glutamate metabolism, glyoxylate and dicarboxylate metabolism, tryptophan metabolism, nitrogen metabolism, and phenylalanine, tyrosine and tryptophan biosynthesis, suggesting a treatment-specific metabolic signature for GSS.

Correlation analysis revealed strong associations between behavioral and cytokine outcomes and specific serum metabolites (Supplementary Table S1). In particular, TST immobility showed robust negative correlations with several amino acid–related metabolites (L-β-homoproline, histidine, threonine, creatinine, phenylpyruvic acid, indolelactic acid and xanthosine), whereas IL-6, IL-1β, and TNF-α exhibited strong positive correlations with propionylcarnitine and indolepyruvic acid.

### Neuroprotective Properties of GSS through Antioxidant Activation and TFEB-Mediated Lysosomal Clearance

To evaluate the neuroprotective effects of GSS on neuronal cells, HT22 cells were exposed to H_2_O_2_ and subsequently treated with GSS at concentrations of 50, 100, and 200 μg/ml (Fig. S3). Significant neuroprotective effects were observed in the groups treated with 100 and 200 μg/ml of GSS compared with the H_2_O_2_-only group.

**Fig. 4:**
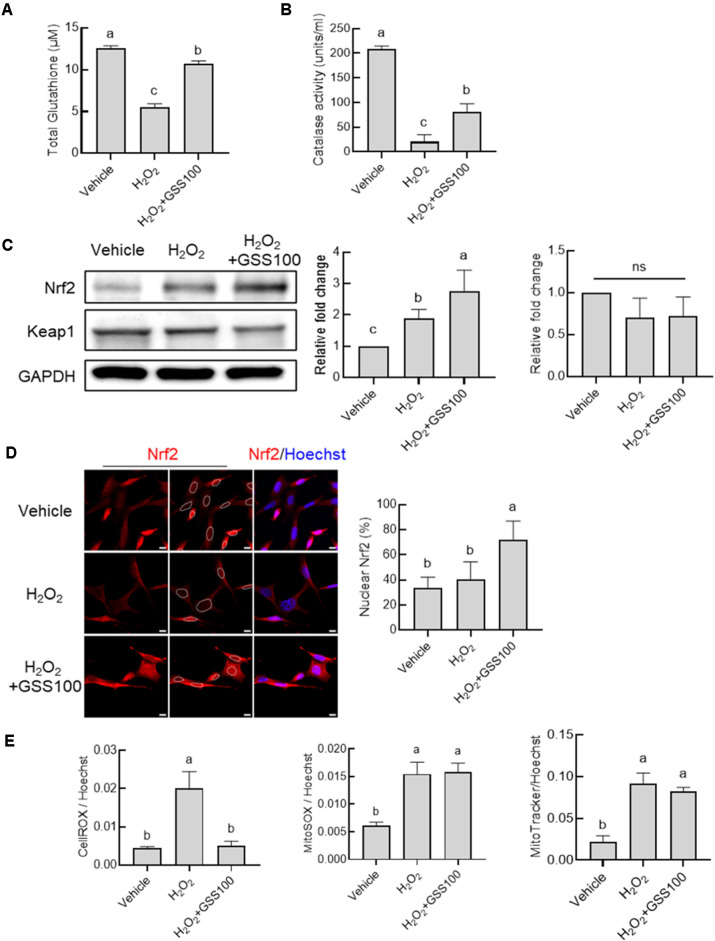
Gami-Soyosan (GSS) protected HT22 cells against oxidative stress via activation of the Nrf2 antioxidant pathway. (**A**) GSS treatment significantly increased intracellular reduced glutathione (GSH) levels, the major cellular antioxidant. (**B**) GSS enhanced catalase activity, a key H_2_O_2_-decomposing enzyme. (**C**) Representative western blot analysis of total cellular Nrf2 and Keap1 protein expression after GSS treatment. GSS significantly upregulated Nrf2 protein levels while Keap1, the negative regulator of Nrf2, was not affected as shown in the plots. GAPDH was used as loading control. (**D**) Immunofluorescence staining and a plot showing nuclear translocation of Nrf2 after GSS treatment (dashed lines indicated the outline the nucleus). Scale bar = 10 µm (**E**) Plots represent intracellular and mitochondrial reactive oxygen species (ROS) levels, as determined by CellROX, MitoSOX and MitoTracker assays, respectively. Data were mean ± SEM from three independent experiments. Statistical analysis was performed using one-way analysis of variance (ANOVA) followed by Tukey’s post hoc test.

To elucidate whether these neuroprotective effects are mediated by antioxidant mechanisms, HT22 cells were treated with GSS in the presence of H_2_O_2_ and assessed for key antioxidant markers. GSS significantly restored H_2_O_2_-induced depletion of intracellular glutathione (Fig. 4A) and markedly enhanced catalase activity, an important H_2_O_2_-scavenging enzyme (Fig. 4B). Nrf2, an upstream regulator of cellular antioxidant pathways, was significantly induced by GSS, whereas Keap1, a negative regulator of Nrf2, was not changed (Fig. 4C). Immunofluorescence staining further revealed prominent nuclear translocation of Nrf2 following GSS treatment, confirming activation of the Nrf2-mediated antioxidant pathway (Fig. 4D). Consistent with these changes, cellular reactive oxygen species (ROS) levels were reduced by GSS, whereas mitochondrial ROS levels were not significantly altered (Fig. 4E), suggesting that GSS primarily modulates whole-cell antioxidant defenses rather than directly affecting mitochondrial ROS.

**Fig. 5:**
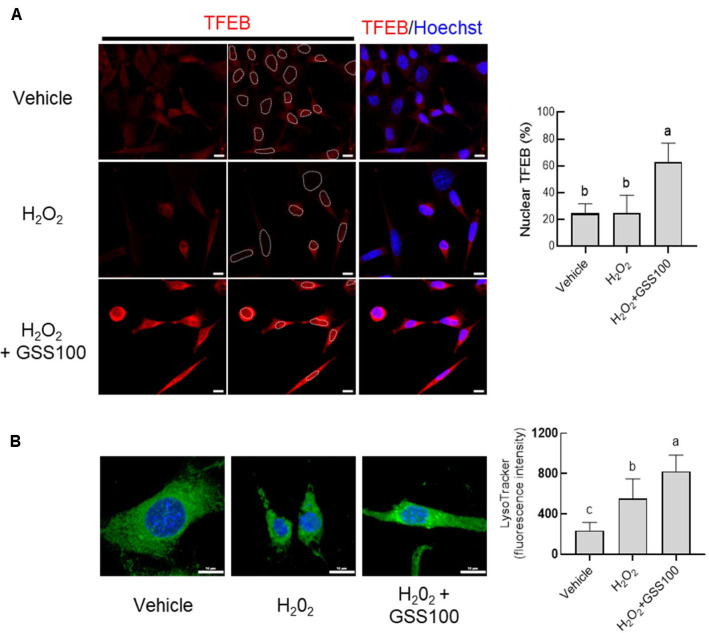
GSS promoted lysosomal function and TFEB nuclear translocation. (**A**) Immunofluorescence staining demonstrated nuclear translocation of TFEB (**B**) Lysosomal activity was assessed by LysoTracker and quantified via fluorescence imaging. Scale bar, 10 μm. Statistical analysis was performed using oneway analysis of variance (ANOVA) followed by Tukey’s post hoc test.

To determine whether GSS exerts additional neuroprotective effects beyond its direct antioxidant activity, we next investigated whether GSS activates the lysosomal–autophagy clearance pathway. GSS treatment led to increased nuclear accumulation of TFEB, a master regulator of autophagy and lysosomal biogenesis (Fig. 5A). In line with this, H_2_O_2_-treated HT22 cells displayed elevated lysosomal activity, consistent with a compensatory response to oxidative stress, and GSS treatment further amplified this lysosomal activity (Fig. 5B). These findings indicate that GSS-driven enhancement of the lysosomal clearance pathway may serve as a key neuroprotective mechanism that acts in concert with its antioxidant effects. Taken together, these results demonstrate that GSS exerts neuroprotective effects against H_2_O_2_-induced oxidative damage through dual mechanisms, namely activation of the Nrf2-dependent antioxidant defense system and enhancement of TFEB-mediated autophagy–lysosomal clearance pathways.

## Discussion

The present study demonstrated that GSS exerts antidepressant-like effects in a CORT-induced depression model through multi-layered mechanisms. Behavioral assessments revealed that GSS significantly reduced immobility in the FST and TST, comparable to the effects of fluoxetine. These behavioral improvements were accompanied by distinct changes in systemic metabolism and direct antioxidative protection in neuronal cells. Together, the findings suggest that GSS may act on interconnected immune, metabolic, and oxidative pathways implicated in stress-related depression.

At the behavioral level, CORT administration reproduced depression-like phenotypes, consistent with previous studies showing that chronic glucocorticoid exposure induces behavioral despair [34, 35]. GSS treatment ameliorated these behaviors, supporting its potential role as a complementary approach for managing depression-like symptoms. Importantly, earlier work has highlighted the involvement of pro-inflammatory cytokines such as IL-6, IL-1β, and TNF-α in the pathophysiology of depression, linking elevated cytokine levels to altered neurotransmission and increased relapse risk [36, 37]. In the present study, GSS not only improved depression-like behaviors but also reduced serum levels of IL-6, IL-1β, and TNF-α. These concomitant changes are consistent with a potential contribution of reduced systemic inflammatory cytokine tone to its behavioral effects, although the specific cellular and molecular pathways underlying this association were not directly examined in the present study.

Metabolomic profiling provided further insights into the systemic effects of GSS. The branched-chain amino acids (BCAAs), arginine, and histidine/β-alanine pathways, which are included in Cluster A metabolites, showed a significant perturbation following CORT exposure and a recovery trend following GSS treatment. This suggests that GSS may help restore amino acid and nitrogen homeostasis under stress. Altered BCAA metabolism has been reported in patients with major depressive disorder (MDD), with reductions in valine, leucine, and isoleucine [38], indicating a perturbation of metabolic balance relevant to synaptic and energy regulation in MDD. Depression impairs arginine metabolism, leading to a lower global arginine bioavailability ratio and associated vulnerability to oxidative stress [39], and GSS stabilized this pathway. Similarly, histidine/β-alanine metabolism contributes to antioxidant defenses, and treatment targeting this pathway has been reported to alleviate depressive symptoms in clinical populations [40]. Cluster A findings suggest that GSS may contribute to the restoration of amino acid and nitrogen metabolic balance, particularly within BCAA, arginine, and histidine/β-alanine pathways, which could be associated with its antidepressant-like effects. However, these observations are based solely on serum metabolomic profiling and should be regarded as hypothesis-generating, and targeted experimental studies will be required to determine whether these amino acid–related pathways play a causal role in the actions of GSS.

Cluster B metabolites, involving taurine and hypotaurine metabolism, primary bile acid biosynthesis, and linoleic acid metabolism, did not return toward the normal pattern but instead showed opposite regulation in both fluoxetine- and GSS-treated groups. This suggests that treatment did not simply restore the pre-stress balance but rather established an alternative metabolic state. Taurine is a key regulator of cellular redox balance and synaptic stability [41]. In a chronic social defeat stress model, taurine supplementation was shown to reverse depressive-like behaviors and to protect against dendritic spine loss in cortical neurons, supporting its role in stress resilience [42, 43]. Similarly, alterations in bile acid composition have been reported in patients with MDD, with significant associations to mood regulation and treatment response through gut–liver–brain signaling pathways. Emerging evidence indicates that bile acids act on central receptors such as FXR and TGR5, thereby modulating neuroinflammatory signaling, neurotransmitter regulation, and energy metabolism [44, 45]. In patients with MDD, specific primary and secondary bile acids have been associated not only with baseline depressive symptom severity but also with differential responses to antidepressant therapy, suggesting their potential role as biomarkers for treatment stratification [46, 47]. Together, these results imply that rather than restoring metabolic parameters to baseline, both GSS and fluoxetine may promote a restructured metabolic state in taurine and bile acid–related pathways.

Cluster C metabolites, encompassing alanine, aspartate and glutamate metabolism, glyoxylate and dicarboxylate metabolism, tryptophan metabolism, nitrogen metabolism, and phenylalanine, tyrosine and tryptophan biosynthesis, demonstrated distinct patterns between fluoxetine and GSS-treated groups. Unlike Cluster A, which showed a tendency toward recovery of amino acid balance, Cluster C indicates that fluoxetine and GSS may engage different metabolic nodes to exert antidepressant effects. Tryptophan metabolism is a particularly relevant pathway in stress-related depression. Under chronic inflammatory conditions, the balance of tryptophan metabolism may shift away from serotonin synthesis toward the kynurenine pathway, leading to increased production of neuroactive metabolites such as kynurenic and quinolinic acid, which have been implicated in altered mood regulation [48-50]. Such alterations highlight the importance of tryptophan pathway regulation as a mechanistic intersection where pharmacological and herbal interventions may diverge. The divergence observed between fluoxetine and GSS implies that while fluoxetine may primarily act through serotonin reuptake inhibition, GSS could modulate broader aspects of tryptophan and related amino acid metabolism. Furthermore, nitrogen and glyoxylate-related pathways have been linked to mitochondrial energy metabolism and oxidative balance. Alterations in these pathways in depression have been reported in metabolomic studies [51-53], and the distinct regulation by GSS versus fluoxetine may highlight differences in how pharmacological versus herbal interventions stabilize cellular energy homeostasis. Taken together, Cluster C underscores that while both fluoxetine and GSS ameliorate depression-like phenotypes, they likely do so through partially non-overlapping metabolic mechanisms. This divergence supports the view that GSS may not merely mimic conventional antidepressants but may instead act via complementary pathways, particularly those involving amino acid tryptophan metabolism.

In HT22 neuronal cells exposed to H_2_O_2_, GSS exerted a dose-dependent neuroprotective effect at 100 and 200 μg/ml, which is consistent with its antioxidant and cellular stress–modulating properties. In line with this, GSS significantly restored H_2_O_2_-induced depletion of intracellular glutathione and enhanced catalase activity, indicating reinforcement of endogenous antioxidant defenses. GSS also increased Nrf2 protein levels and promoted its nuclear translocation, whereas Keap1 expression remained unchanged, supporting activation of an Nrf2-dependent antioxidant pathway. These changes were accompanied by a reduction in whole-cell ROS levels, while mitochondrial superoxide production and mitochondrial content were not significantly altered, suggesting that GSS primarily modulates global cellular redox balance rather than directly affecting mitochondrial ROS. In our HT22 cell experiments, H_2_O_2_ treatment alone increased mitochondrial content compared with vehicle controls, consistent with previous reports that oxidative stress can induce a compensatory increase in mitochondrial mass as an adaptive response rather than a simple marker of damage [54]. At the same time, GSS increased nuclear accumulation of TFEB and further augmented lysosomal activity beyond the H_2_O_2_-induced compensatory response, consistent with activation of the autophagy–lysosome pathway that enhances cellular clearance capacity and strengthens neuronal resistance to oxidative stress [55, 56]. Lysosomes are also known to function as key regulators of amino acid metabolism and nutrient sensing [57], providing a potential link between the cellular effects of GSS and the systemic changes in amino acid and nitrogen metabolism observed in our serum metabolomic analysis. Moreover, activation of autophagy–lysosome pathways has been reported to limit inflammasome activity and reduce IL-1β maturation, thereby suppressing pro-inflammatory cytokine production [58]. Although we did not directly assess inflammasome components or IL-1β processing in this study, these prior findings provide a plausible link between TFEB–lysosome activation and the reduced circulating IL-6, IL-1β, and TNF-α levels observed after GSS administration. Thus, our data support a model in which lysosomal and antioxidant mechanisms may act in parallel with systemic metabolic regulation to contribute to the antidepressant-like effects of GSS, but the precise anti-inflammatory pathways remain to be elucidated.

In light of these findings, the metabolomic, inflammatory, and lysosomal data can be interpreted within a unified framework. Systemically, GSS partially normalized amino acid- and nitrogen-related pathways in Cluster A and induced an alternative metabolic state in taurine, hypotaurine, and bile acid–related pathways in Clusters B and C. Lysosomes are increasingly recognized as central hubs for nutrient sensing and amino acid recycling that coordinate cellular metabolism and stress adaptation through TFEB-dependent regulation of autophagy and lysosomal biogenesis [55, 57]. In our HT22 experiments, GSS-induced activation and nuclear translocation of TFEB, together with enhanced lysosomal activity, are therefore consistent with a cellular response that increases degradative and recycling capacity under oxidative stress [55, 57]. Autophagy–lysosome activation has also been shown to limit inflammasome activity and IL-1β production, thereby restraining downstream inflammatory cytokine signaling [58]. In parallel, GSS reduced circulating IL-6, IL-1β, and TNF-α *in vivo*, cytokines that are known to contribute to the development and maintenance of depressive symptoms [36, 37]. Taken together, these converging observations support an integrated model in which GSS modulates amino acid and bile acid metabolism, reduces systemic pro-inflammatory cytokine levels, and enhances TFEB-associated lysosomal clearance. These processes may act in concert to contribute to the antidepressant-like phenotype in the CORT-induced model, although the individual causal contributions of each pathway remain to be determined.

While this study provides novel insights into the antidepressant-like effects of GSS, several limitations should be considered. First, the amino acid–related metabolic pathways identified in our serum metabolomic analysis were not functionally validated, so these findings should be regarded as exploratory and confirmed in future studies using targeted metabolite and protein assays. Second, although GSS reduced circulating IL-6, IL-1β, and TNF-α levels, we measured only a limited cytokine panel and did not investigate upstream inflammatory signaling pathways (*e.g.*, NF-κB or inflammasome components). Third, although we observed GSS-induced TFEB nuclear translocation and enhanced lysosomal activity in HT22 cells, we did not assess autophagy/lysosomal markers such as LC3-II and p62 or evaluate these pathways *in vivo*, so the contribution of autophagy–lysosome modulation to the antidepressant-like effects of GSS remains to be clarified. Taken together, the immune, metabolic, and lysosomal mechanisms discussed in this manuscript should be regarded as hypothesis-generating, yet the integrated behavioral, cytokine, and metabolomic data still support GSS as a promising complementary strategy for managing depression-like symptoms.

## Conclusion

The aim of this study was to evaluate the antidepressant-like effects of GSS in a corticosterone-induced mouse model of depression. Behavioral assessments, serum cytokine analysis, metabolomic profiling, and neuronal cell experiments were conducted to investigate its therapeutic potential and underlying mechanisms. GSS improved depression-like behaviors, reduced systemic pro-inflammatory cytokines, modulated amino acid and nitrogen metabolic pathways, and conferred neuronal protection by enhancing lysosomal activity and attenuating oxidative stress. These findings highlight the multi-layered actions of GSS across behavioral, immune, and metabolic domains and support its potential as a complementary therapeutic approach for stress-related depression.

## Supplemental Materials

Supplementary data for this paper are available on-line only at http://jmb.or.kr.



## References

[1] Nabavi SM, Daglia M, Braidy N, Nabavi SF (2017). Natural products, micronutrients, and nutraceuticals for the treatment of depression: a short review. Nutr. Neurosci..

[2] Kennedy SH, Andersen HF, Thase ME (2009). Escitalopram in the treatment of major depressive disorder: a meta-analysis. Curr. Med. Res. Opin..

[3] Cipriani A, Furukawa TA, Salanti G, Geddes JR, Higgins JPT, Churchill R (2009). Comparative efficacy and acceptability of 12 new-generation antidepressants: a multiple-treatments meta-analysis. Lancet.

[4] Hansen RA, Gartlehner G, Lohr KN, Gaynes BN, Carey TS (2005). Efficacy and safety of second-generation antidepressants in the treatment of major depressive disorder. Ann. Intern. Med..

[5] Entsuah AR, Huang H, Thase ME (2001). Response and remission rates in different subpopulations with major depressive disorder administered venlafaxine, selective serotonin reuptake inhibitors, or placebo. J. Clin. Psychiatry.

[6] Zhuang W, Liu SL, Xi SY, Feng YN, Wang K, Abduwali T (2023). Traditional Chinese medicine decoctions and Chinese patent medicines for the treatment of depression: efficacies and mechanisms. J. Ethnopharmacol..

[7] Peng S, Zhou Y, Lu M, Wang Q (2022). Review of herbal medicines for the treatment of depression. Nat. Prod. Commun..

[8] Li C, Huang B, Zhang YW (2021). Chinese herbal medicine for the treatment of depression: effects on the neuroendocrine-immune network. Pharmaceuticals.

[9] Lin SH, Chou ML, Chen WC, Lai YS, Lu KH, Hao CW (2015). A medicinal herb, *Melissa officinalis* L. ameliorates depressive-like behavior of rats in the forced swimming test via regulating the serotonergic neurotransmitter. J. Ethnopharmacol..

[10] Muszyńska B, Łojewski M, Rojowski J, Opoka W, Sułkowska-Ziaja K (2015). Natural products of relevance in the prevention and supportive treatment of depression. Psychiatr. Pol..

[11] Lee S, Rhee DK (2017). Effects of ginseng on stress-related depression, anxiety, and the hypothalamic-pituitary-adrenal axis. J. Ginseng Res..

[12] Wang YS, Shen CY, Jiang JG (2019). Antidepressant active ingredients from herbs and nutraceuticals used in TCM: pharmacological mechanisms and prospects for drug discovery. Pharmacol. Res..

[13] Shen CY, Jiang JG, Yang L, Wang DW, Zhu W (2017). Anti-ageing active ingredients from herbs and nutraceuticals used in traditional Chinese medicine: pharmacological mechanisms and implications for drug discovery. Br. J. Pharmacol..

[14] Park DM, Kim SH, Park YC, Kang WC, Lee SR, Jung IC (2014). The comparative clinical study of efficacy of *Gamisoyo-San* (*Jiaweixiaoyaosan*) on generalized anxiety disorder according to differently manufactured preparations: multicenter, randomized, double-blind, placebo-controlled trial. J. Ethnopharmacol..

[15] Ministry of Health and Welfare (Korea). 2009. Revised notification of national health service medical care benefit standard.

[16] Kim JY, Kwak DH, Ju EJ, Kim SM, Lee DH, Keum KS (2004). Effects of Gamisoyosan on *in vitro* fertilization and ovulation of stressed mice by electric shock. Arch. Pharm. Res..

[17] Lee SH, Lee JM, Cho JH, Jang JB, Lee KS (2010). Antioxidant and neuroprotective effects of Gamisoyo-san. J. Orient. Obstet. Gynecol..

[18] Kang BC, Sung KH, Song IH, Kim UC, Kwon DI, Park KH (2004). The clinical review on three cases of UL-syndrome (*Uljung*) induced by chronic stress. Korean J. Orient. Int. Med..

[19] Alarcon-Barrera JC, Kostidis S, Ondo-Mendez A, Giera M (2022). Recent advances in metabolomics analysis for early drug development. Drug Discov. Today.

[20] Pu J, Liu Y, Gui S, Tian L, Yu Y, Song X (2021). Metabolomic changes in animal models of depression: a systematic analysis. Mol. Psychiatry.

[21] Zhao M, Ren Z, Zhao A, Tang Y, Kuang J, Li M (2024). Gut bacteria-driven homovanillic acid alleviates depression by modulating synaptic integrity. Cell Metab..

[22] Song AQ, Gao B, Fan JJ, Zhu YJ, Zhou J, Wang YL (2020). NLRP1 inflammasome contributes to chronic stress-induced depressive-like behaviors in mice. J. Neuroinflammation.

[23] Slattery DA, Cryan JF (2012). Using the rat forced swim test to assess antidepressant-like activity in rodents. Nat. Protoc..

[24] Li E, Yin H, Su M, Li Q, Zhao Y, Zhang L (2023). Inhibition of ferroptosis alleviates chronic unpredictable mild stress-induced depression in mice via tsRNA-3029b. Brain Res. Bull..

[25] Pang Z, Lu Y, Zhou G, Hui F, Xu L, Viau C (2024). MetaboAnalyst 6.0: towards a unified platform for metabolomics data processing, analysis and interpretation. Nucleic Acids Res..

[26] O'Neil MF, Moore NA (2003). Animal models of depression: are there any?. Hum. Psychopharmacol..

[27] Ardayfio P, Kim K-S (2006). Anxiogenic-like effect of chronic corticosterone in the light-dark emergence task in mice. Behav. Neurosci..

[28] David DJ, Samuels BA, Rainer Q, Wang JW, Marsteller D, Mendez I (2009). Neurogenesis-dependent and -independent effects of fluoxetine in an animal model of anxiety/depression. Neuron.

[29] Steru L, Chermat R, Thierry B, Simon P (1985). The tail suspension test: a new method for screening antidepressants in mice. Psychopharmacology.

[30] Xie X, Shen Q, Yu C, Xiao Q, Zhou J, Xiong Z (2020). Depression-like behaviors are accompanied by disrupted mitochondrial energy metabolism in chronic corticosterone-induced mice. J. Steroid Biochem. Mol. Biol..

[31] Zhang K, Yang J, Wang F, Pan X, Liu J, Wang L (2016). Antidepressant-like effects of Xiaochaihutang in a neuroendocrine mouse model of anxiety/depression. J. Ethnopharmacol..

[32] Kulkarni SK, Dhir A (2007). Effect of various classes of antidepressants in behavioral paradigms of despair. Prog. Neuropsychopharmacol. Biol. Psychiatry.

[33] Kraeuter AK, Guest PC, Sarnyai Z. 2019. The open field test for measuring locomotor activity and anxiety-like behavior, pp. 99-103. *In* Guest PC (ed.), Pre-Clinical Models: Techniques and Protocols. Methods Mol. Biol. 1916. Humana Press (Springer), New York, NY. 10.1007/978-1-4939-8994-2_9 30535687

[34] Shoji H, Maeda Y, Miyakawa T (2024). Chronic corticosterone exposure causes anxiety- and depression-related behaviors with altered gut microbial and brain metabolomic profiles in adult male C57BL/6J mice. Mol. Brain.

[35] Dwivedi Y, Roy B, Lugli G, Rizavi H, Zhang H, Smalheiser NR (2015). Chronic corticosterone-mediated dysregulation of microRNA network in prefrontal cortex of rats: relevance to depression pathophysiology. Transl. Psychiatry.

[36] Felger JC, Lotrich FE (2013). Inflammatory cytokines in depression: neurobiological mechanisms and therapeutic implications. Neuroscience.

[37] Hassamal S (2023). Chronic stress, neuroinflammation, and depression: an overview of pathophysiological mechanisms and emerging anti-inflammatories. Front. Psychiatry.

[38] Baranyi A, Amouzadeh-Ghadikolai O, von Lewinski D, Rothenhäusler H-B, Theokas S, Robier C (2016). Branched-chain amino acids as new biomarkers of major depression-a novel neurobiology of mood disorder. PLoS One.

[39] Ali-Sisto T, Tolmunen T, Viinamäki H, Mäntyselkä P, Valkonen-Korhonen M, Koivumaa-Honkanen H (2018). Global arginine bioavailability ratio is decreased in patients with major depressive disorder. J. Affect. Disord..

[40] Kabthymer RH, Saadati S, Lee M, Hariharan R, Feehan J, Mousa A (2025). Carnosine/histidine-containing dipeptide supplementation improves depression and quality of life: systematic review and meta-analysis of randomized controlled trials. Nutr. Rev..

[41] Schaffer S, Kim HW (2018). Effects and mechanisms of taurine as a therapeutic agent. Biomol. Ther..

[42] Zhu Y, Wang R, Fan Z, Luo D, Cai G, Li X (2023). Taurine alleviates chronic social defeat stress-induced depression by protecting cortical neurons from dendritic spine loss. Cell. Mol. Neurobiol..

[43] Li Y, Li L, Wei S, Yao J, Liang B, Chu X (2024). Integrating transcriptomics and metabolomics to elucidate the mechanism by which taurine protects against DOX-induced depression. Sci. Rep..

[44] Ye D, He J, He X (2024). The role of bile acid receptor TGR5 in regulating inflammatory signalling. Scand. J. Immunol..

[45] McMillin M, DeMorrow S (2016). Effects of bile acids on neurological function and disease. FASEB J..

[46] Corrivetti G, Monaco F, Vignapiano A, Marenna A, Panarello E, Gruttola BD (2025). Precision Medicine for Depression: improving treatment response and remission. Asian J. Psychiatr..

[47] Dunlop BW, Cha J, Mayberg HSM, Choi KS, Craighead WE, Dehkordi SM, *et al*. 2024. Association of bile acids with connectivity of executive control and default mode networks in patients with major depression. bioRxiv: 2024.12.20.629637. https://doi.org/10.1101/2024.12.20.629637. 10.1101/2024.12.20.629637

[48] Sakurai M, Yamamoto Y, Kanayama N, Hasegawa M, Mouri A, Takemura M (2020). Serum metabolic profiles of the tryptophan-kynurenine pathway in the high-risk subjects of major depressive disorder. Sci. Rep..

[49] Sha Q, Madaj Z, Keaton S, Escobar Galvis ML, Smart L, Krzyzanowski S (2022). Cytokines and tryptophan metabolites can predict depressive symptoms in pregnancy. Transl. Psychiatry.

[50] Correia AS, Vale N (2022). Tryptophan metabolism in depression: a narrative review with a focus on serotonin and kynurenine pathways. Int. J. Mol. Sci..

[51] Zhao J, Jung YH, Jin Y, Kang S, Jang CG, Lee J (2019). A comprehensive metabolomics investigation of hippocampus, serum, and feces affected by chronic fluoxetine treatment using the chronic unpredictable mild stress mouse model of depression. Sci. Rep..

[52] Pu J, Liu Y, Gui S, Tian L, Yu Y, Wang D (2022). Effects of pharmacological treatment on metabolomic alterations in animal models of depression. Transl. Psychiatry.

[53] Gao Y, Hu JZ, Wen ZP, Dong T, Du XZ, Liu ZF (2025). Metabolomic insights into late-life depression: a systematic review. BMC Geriatr..

[54] Lee CF, Liu CY, Hsieh, RH, Wei YH. 2005. Oxidative stress-induced depolymerization of microtubules and alteration of mitochondrial mass in human cells. *Ann. N. Y. Acad. Sci.* **1042:** 246-254. https://doi.org/10.1196/annals.1338.027. 10.1196/annals.1338.027 15965069

[55] Settembre C, Di Malta C, Polito VA, Arencibia MG, Vetrini F, Erdin S (2011). TFEB links autophagy to lysosomal biogenesis. Science.

[56] Zhuang XX, Wang SF, Tan Y, Song JX, Zhu Z, Wang ZY (2020). Pharmacological enhancement of TFEB-mediated autophagy alleviated neuronal death in oxidative stress-induced Parkinson's disease models. Cell Death Dis..

[57] Ballabio A, Bonifacino JS (2020). Lysosomes as dynamic regulators of cell and organismal homeostasis. Nat. Rev. Mol. Cell Biol..

[58] Shi CS, Shenderov K, Huang NN, Kabat J, Abu-Asab M, Fitzgerald KA (2012). Activation of autophagy by inflammatory signals limits IL-1β production by targeting ubiquitinated inflammasomes for destruction. Nat. Immunol..

