# Supplementary material for: Multi-Layered Antidepressant Mechanisms of Gami-Soyosan in a Corticosterone-Induced Mouse Model
Source: J Microbiol Biotechnol. 2026 Jan 26;36:e2509033. doi: 10.4014/jmb.2509.09033 (PMC12868945; doi:10.4014/jmb.2509.09033)
Supplement: Supplementary file 1 [file jmb-36-e2509033-supple.pdf]

J. Microbiol. Biotechnol. 2026. 36: e2509033
https://doi.org/10.4014/jmb.2509.09033

Supplementary Figures and Table

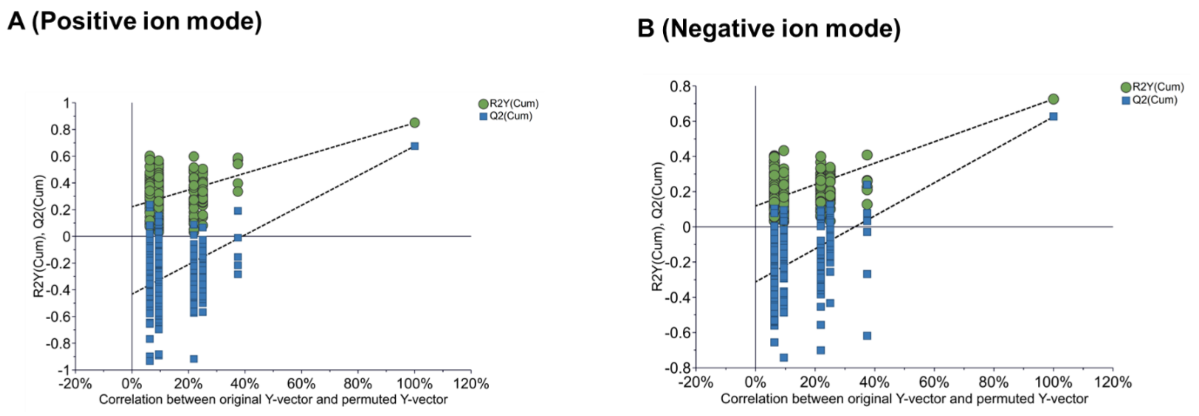

Fig. S1. Permutation test results (n = 200) for evaluating the reliability of the orthogonal partial least squares discriminant analysis (OPLS-DA) models. **(A)** Positive ion mode, **(B)** Negative ion mode. The results confirmed that overfitting did not occur in both models.

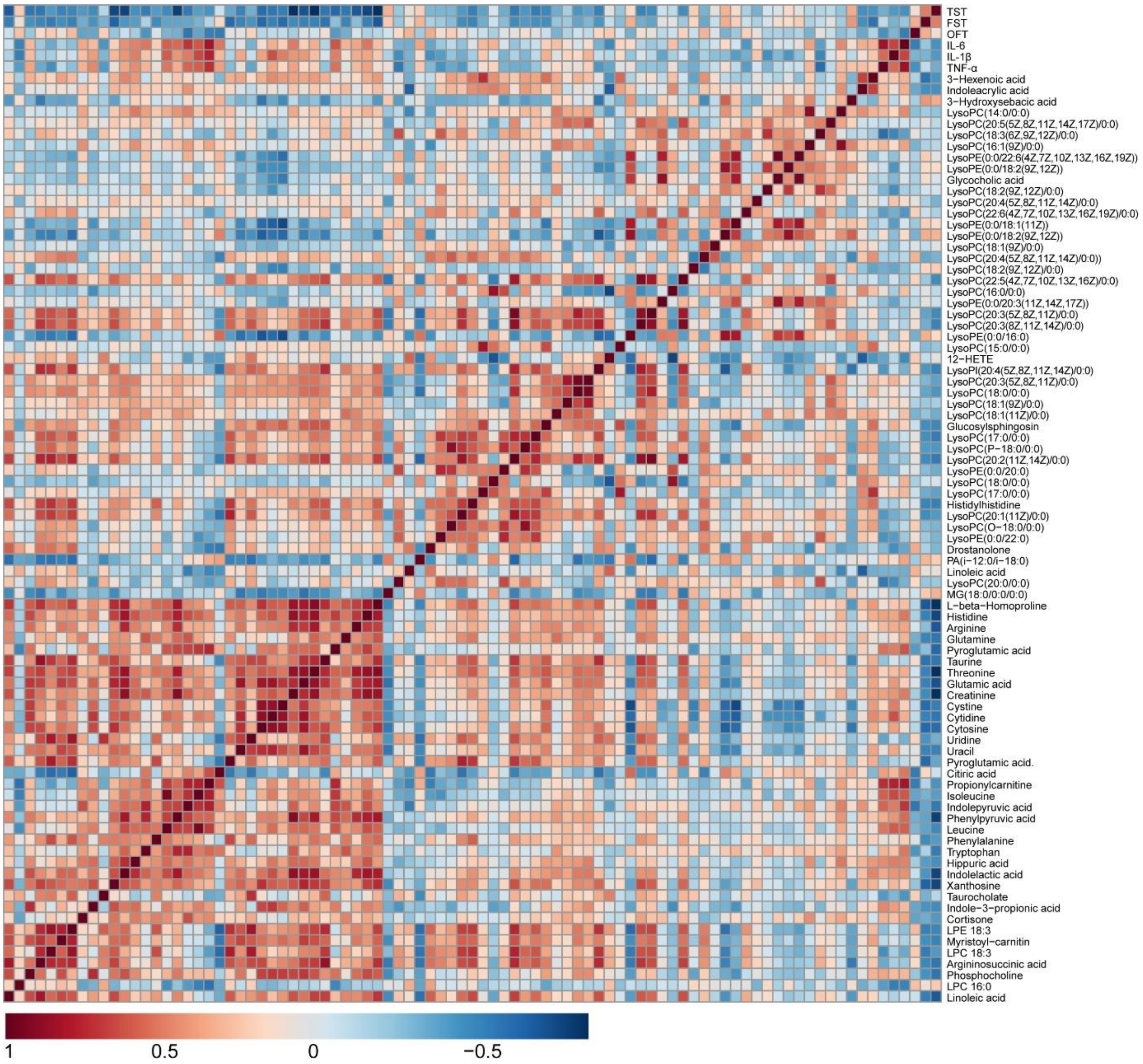

Fig. S2. **Correlation heatmap integrating behavioral outcomes, serum cytokines, and serum metabolites in CORT-induced mice.** The heatmap displays Spearman's rank correlation coefficients between behavioral measures (immobility time in the FST and TST, locomotor activity in the OFT), serum cytokine levels (IL-6, IL-1β, and TNF-α), and normalized serum metabolite intensities across all experimental groups. All variables were autoscaled before analysis, and correlations were visualized as a clustered heatmap, with warm colors indicating positive correlations and cool colors indicating negative correlations.

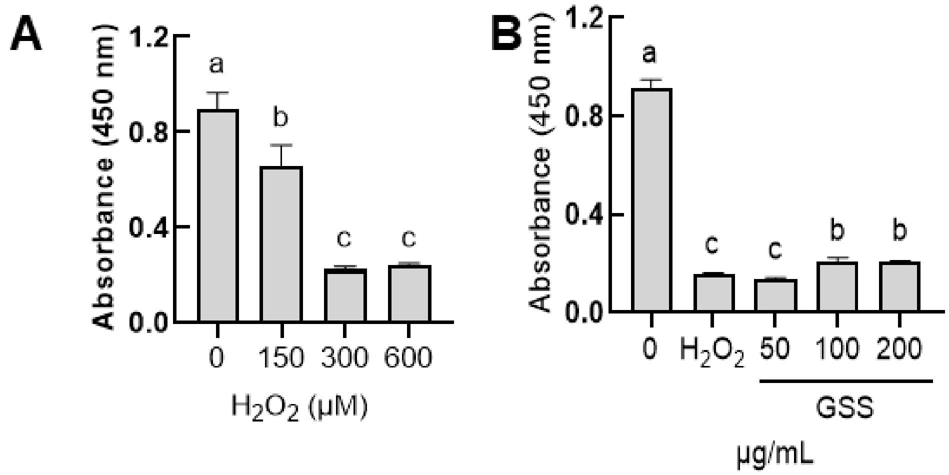

Fig. S3. Effects of H_2O_2 and Gami-Soyosan (GSS) on HT22 cell viability.

(A) Cell viability of HT22 cells after exposure to increasing concentrations of H_2O_2 for 24 h. **(B)** Changes in HT22 cell viability following co-treatment with H_2O_2 and GSS at the indicated concentrations. Data are expressed as mean ± SEM from three independent experiments. Different letters above bars indicate statistically significant differences between groups ($p < 0.05$, one-way ANOVA followed by Tukey's post hoc test).

Table S1. **Correlations between behavioral and cytokine outcomes and serum metabolite markers in CORT-induced mice.**

Behavioral / Cytokine variable	Correlated metabolite	Correlation coefficient	Significance
TST immobility	L-β-Homoproline	-0.7999	***
	Histidine	-0.7203	***
	Threonine	-0.8125	***
	Creatinine	-0.8025	***
	Phenylpyruvic acid	-0.7466	***
	Indolelactic acid	-0.7327	***
	Xanthosine	-0.7155	***
IL-6 (Serum)	Propionyl-carnitine	0.8276	***
	Indolepyruvic acid	0.7109	***
IL-1β (Serum)	Propionyl-carnitine	0.7664	***
TNF-α (Serum)	Propionyl-carnitine	0.7341	***

Correlation pairs with an absolute Spearman correlation coefficient ≥ 0.7 and an FDR-adjusted p-value < 0.05 are summarized (*, $p < 0.05$; **, $p < 0.01$; ***, $p < 0.001$).
